# Fabrication of Triple-parted Stomata-inspired Membrane with Stimulus-responsive Functions

**DOI:** 10.1038/srep21258

**Published:** 2016-02-18

**Authors:** Hyejeong Kim, Sang-Joon Lee

**Affiliations:** 1 Center for Biofluid and Biomimic Research, Department of Mechanical Engineering, Pohang University of Science and Technology (POSTECH), Pohang, 790-784, South Korea

## Abstract

Hydrogels with controllable morphologies and functional movements present a wide range of practical applications. In this work, a triple-parted stomata-inspired membrane (SIM) was fabricated using a UV light cured hydrogel by polymerization-induced diffusion of reactants. A single UV light illumination yielded the SIM that has completely-penetrating pores and semi-penetrated parts. Membranes of various shapes can be easily fabricated within a few minutes by changing the photomask design and composition of the pre-gel solution. Similar to stomatal movement, pores in the fabricated SIM open and close their aperture in response to thermal stimuli. The deformability and transparency of the SIM can be easily controlled for a given application. This SIM exhibits stimulus-response, and therefore has numerous practical applications, such as filter membranes with self-adjustable pores, membrane-based sensors, and functional smart membranes.

The programmed movement of soft materials is inevitably required for smart design of actuators, valves, microlens arrays, microfluidics, polymer brushes, and drug delivery^1^,^2^,^3^,^4^,^5^,^6^,^7^,^8^. As such, hydrogels that respond to various stimuli such as pH, humidity, temperature, electric/magnetic field, light, and ion strength have been developed^9^,^10^,^11^,^12^,^13^. Several of these stimulus-responsive hydrogels have been patterned using lithography^6^,^7^,^14^. Movable functions of these hydrogels have been achieved by adopting various types of surface instability (e.g., wrinkling, creasing, buckling)^6^,^9^,^15^,^16^,^17^, self-folding origami^18^,^19^,^20^,^21^, or bilayer bending^22^,^23^,^24^. Hydrogels with dynamic functions present potential in many practical applications. For use of hydrogels to be practical in the applications, smart design and easy methods to fabricate stimulus-responsive hydrogel structures are required^4^.

For innovative applications of a stimulus-responsive hydrogel, a stomata-inspired membrane (SIM) with temperature-responsive variation of morphology and optical transparency is developed in this study. In our previous study, we fabricated a double-parted SIM through photopolymerization patterning^25^. However, the membrane contains double parts only and therefore has technical limitations for various engineering applications. The physical properties of the fabricated SIM must be investigated in detail to elucidate its functional mechanism. Stomata in plant leaves are small pores, and they are opened and closed by guard cells that contract or swell in response to turgor pressure. The guard cells respond to environmental conditions, (e.g., temperature, light intensity, intercellular CO_2_ concentration) in a multi-sensitive manner. The apertures of the pores exhibit flexible heterogeneity, called stomatal patchiness^26^,^27^. Several biomimetic artificial leaves have been developed based on the functional features of plant leaves^28^,^29^,^30^,^31^,^32^, but heterogeneous responses of stomatal opening or closing have not been achieved.

In this study, we mimic the novel motile movement of guard cells by using a temperature-responsive polymer, and the pores in the SIMs are analogous to the stomata. Conventional methods used to pattern microstructures involve multiple steps, so the cost and time required are high^7^,^14^. An inexpensive and simple effective photopolymerization patterning method is used to fabricate the SIMs. A triple-parted membrane can be fabricated in a single-step UV light illumination at a well-controlled crosslinking level by effectively utilizing polymerization-induced diffusion. During polymerization, reactants in a pre-gel solution diffuse in opposite directions in response to chemical potential gradients. This diffusion process can create three distinct parts of a membrane with pores. When triggered by slight temperature changes, the SIM shrinks to open the pores, similar to guard cells. The main advantage of this proposed approach from our prior study on double-parted membrane formation is the full utilization of the role of radially diffused free radicals to obtain a very thin third part of the membrane^25^. Each part exhibits different degrees of mechanical deformability and transparency upon thermal stimulation. In addition, the morphological configuration and optical properties of SIM can be easily modified for a specific application by changing photomask design or using different compositions of pre-gel solution. A triple-parted SIM, which exhibits stimulus-responsive functions and can be easily fabricated, has numerous practical applications, including filter membranes with self-adjustable pore sizes, membrane-based sensors, smart valves, and membrane-based novel actuators.

## Results and Discussion

### Fabrication of triple-parted SIMs

**Figure 1:**
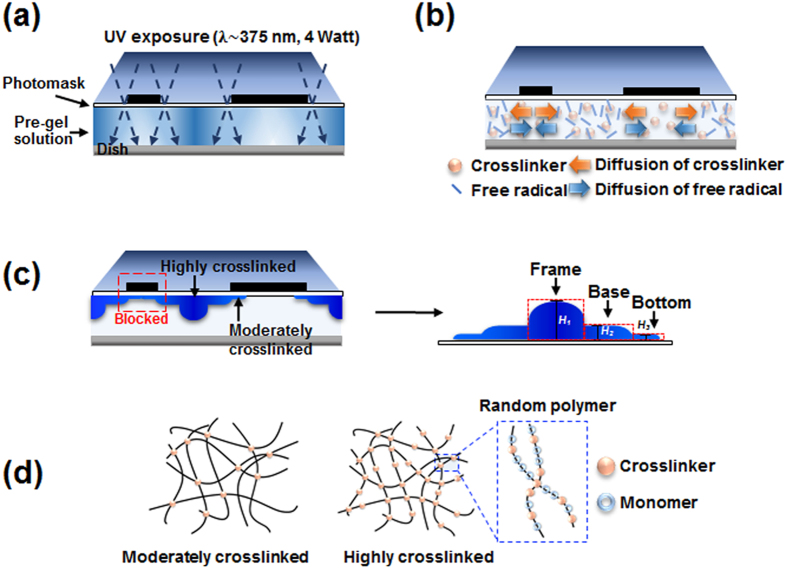
Schematic of the photopolymerization of UV-curable PNIPAAm membrane. (**a)** A pre-gel solution is injected into the space between a photomask and a dish, then irradiated with UV light through the photomask. (**b)** The crosslinker and free radicals diffuse to the opposite directions depending on chemical potential gradient. (**c)** The relatively thick frame is formed at the middle of the exposed area, the thinner base part is built around the frame, and the thinnest bottom part is formed under each pattern. When the pattern size is small, the bottom part can block the pore. (**d)** The frame part is highly crosslinked, and the other parts are comparatively moderately crosslinked.

A UV-curable PNIPAAm hydrogel was used for polymerization-induced diffusion of an irradiated surface of several hundred micrometers. A pre-gel solution was illuminated with UV light through a photomask to fabricate photo-patterned hydrogel membranes. Free radicals generated in the area exposed to UV light cause concurrent formation of two opposite chemical gradients. The radicals initiate polymerization of monomers and crosslinkers (Fig. 1b); the rapid consumption of these species generates a concentration gradient of them; as a result they diffuse from the surrounding area toward the center of the exposed area (Fig. 1b). Formation of free radicals itself induces development of a concentration gradient of them, so they diffuse away from the site of formation into the unexposed area and initiate polymerization there (Fig. 1b). A few free radicals are generated in those areas because UV light is difficult to block. This diffusion of crosslinkers and free radicals opposite directions results in formation of a porous three-part membrane within the polymer membrane.

Ultimately this process generates a relatively thick part (frame) at the center of the UV-exposed area; a thin base around the frame, with the thinnest bottom part under each mask pattern (Fig. 1c). When the pattern is small, the bottom part can block the pore. The randomly-crosslinked polymer membrane attaches covalently to the photomask substrate during polymerization^33^,^34^. Because the monomers and crosslinkers diffuse toward the center of the membrane, the frame is a highly crosslinked polymer and the other parts are comparatively less crosslinked (Fig. 1c,d)^35^.

### Effect of crosslinker concentration

The concentration ratio of monomers and crosslinker determines the crosslinking density of the polymer (Fig. 1d). Therefore the effect of crosslinker concentration on the morphology of the fabricated SIMs was analyzed. Five pre-gel solutions with CR 1–3 were used to prepare SIMs (Table S1). These solutions contained identical amounts of monomer and photoinitiator but different amounts of crosslinker.

**Figure 2:**
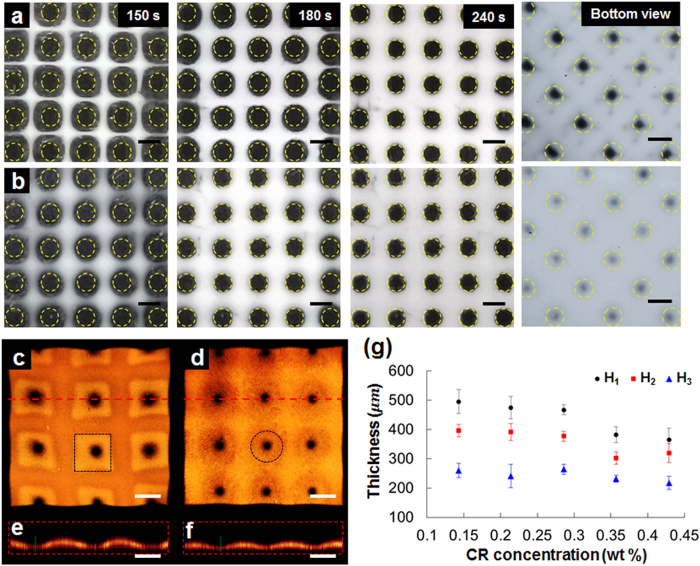
Effect of crosslinker concentration on the morphological features of two SIMs. **(a,b)** Sequential images showing the UV-polymerization procedure of SIMs fabricated with (**a**) CR 1 and (**b**) CR 3 pre-gel solutions. Dotted circles: mask patterns to protect UV irradiation. **(c,d)** Confocal microscopic images of the fabricated SIMs in (**a**,**b**), respectively. **(e,f)** Cross-sectional images of the SIMs extracted from the horizontal dotted line in (**c**,**d**), respectively. **(g)** Variations in the average thickness of the frame (*H*_*1*_), base (*H*_*2*_), and bottom (*H*_*3*_) parts. Scale bar: 1 mm.

Figure 2(a) shows the optical images of the SIMs polymerized with CR 1 and CR 3 pre-gel solutions, wherein the weight percentages of the crosslinker in the pre-gel solution are 0.14 and 0.43, respectively. The images were captured from the top of the photomask. The dotted circles with 800 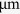 diameter indicate mask patterns where UV light cannot directly reach the polymer membrane. After 150 s of UV light exposure, the middle portions between the mask patterns became opaque because the transparent pre-gel solution had moderately polymerized and a lattice frame had gradually formed. With the CR 1 solution, a thin frame with a clear edge was formed, whereas from with the CR 3 solution, a wide frame with a blurred edge was formed. After 180 s of polymerization, with the CR 1 solution, some regions remained un-polymerized, whereas with the CR 3 solution, most of the exposed area was polymerized. After 240 s of UV light exposure, the membrane with CR 1 solution was almost filled with opaque polymer. These results indicate that high crosslinker concentrations leads to a high degree of polymerization because the effective collision between the reactants increases during the same reaction time^36^,^37^.

Although each pre-gel solution was identically illuminated with UV light for 4 min, the fabricated SIMs exhibit varied polymerization features (bottom view images). Overall, the size of visible pores decreased as crosslinker concentration increased. This trend implies that polymerization propagates toward the unexposed regions during UV light exposure. During polymerization, the local viscosity of the reacting mixture increases, so the mobility of active chains decreases and the termination reactions slow down. This phenomenon, called the gel effect^35^, induces the terminated polymer chains to propagate to a wider region in the membrane during the same period of polymerization.

The fabricated SIM samples are fully swelled in their confocal images. Fig. 2(c,d) show the top view of the reconstructed 3D images of waffle-shaped SIMs fabricated with CR 1 and CR 3 pre-gel solutions. In the SIM fabricated using CR 1 solution, the interface between the frame and the base was distinct, and square base parts are formed. In the SIM with CR 3 solution, the base parts were circular.

Figure 2(e,f) present the cross-sectional images of SIMs extracted from the horizontal dotted line in Fig. 2(c,d). For the SIM with CR 1 solution, the surface exhibits an indented curve along the frame to the base, but when the SIM with CR 3 solution was used, the curve was relatively gentle. The border between the frame and the base became increasingly blunt as crosslinker concentration increased. The average thicknesses of the frame base decreased linearly as crosslinker concentration increased, whereas that of the bottom did not change noticeable (Fig. 2g).

### Effect of photomask pattern size

**Figure 3:**
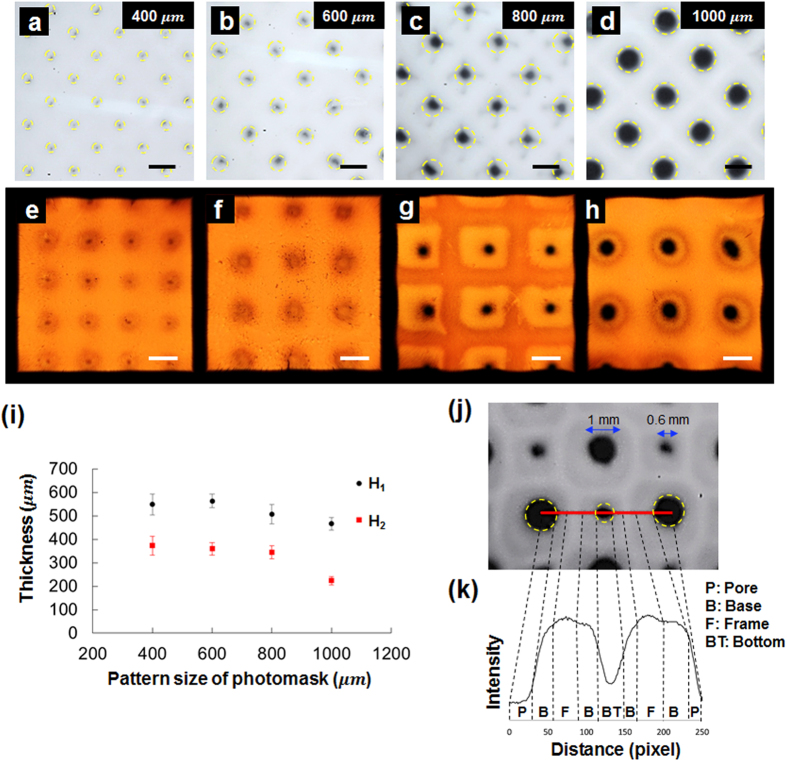
Effect of photomask pattern size (pps) on the morphological features of the fabricated SIMs. **(a–d)** Optical images of the SIMs fabricated with pps of 400, 600, 800, and 1000 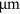 diameter, respectively. **(e–h)** Corresponding 3D images of the SIMs in (**a**–**d**). **(i)** Variations in the average thickness of the frame (*H*_*1*_) and base (*H*_*2*_) parts. **(j)** Special SIM fabricated with a photomask having dual pattern sizes of 1 and 0.6 mm. With single UV light illumination, the frame, base, bottom, and pore parts are fabricated simultaneously in one membrane. **(k)** Each part is clearly distinguished based on the inflection points of light intensity across the membrane. Scale bar: 1 mm.

Chemical reactions in areas that are not exposed to UV light may affect the morphological features of the SIMs. To quantify this effect, photomasks with four different photomask pattern sizes (pps) were used to fabricate SIMs; in all cases, the gap between adjacent patterns was 1 mm, and CR 1 pre-gel solution was used. Figure 3(a–d) show the optical images of SIMs fabricated with pps of 400, 600, 800, and 1000 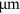 in diameter, respectively. The dotted circles indicate the regions of the mask patterns that are not directly irradiated by UV light. With pps = 400 and 600 μm the pores were almost blocked, and the remaining pores had average diameters of 294 ± 31 μm (Fig. 3a,b); with pps = 800 and 1000 μm the pores were partially blocked, and the remaining pores had average diameter 546 ± 51 μm (Fig. 3c,d). The estimated average propagated length of the polymer chains after UV light exposure for 4 min was 242 ± 24 μm.

The shapes of the frame and base differed among the SIMs (Fig. 3e-h). Figure 3(e-h) show the top view of the reconstructed 3D images of the corresponding SIMs in Fig. 3(a–d). SIM with pps = 400 μm forms cracker-shaped with dots at the corresponding position of the photomask patterns. In the SIM with pps = 600 μm, the pores were also imperfect, and had relatively wide flat bottoms. For these two cases, the small pps allowed the polymerization process to propagate to completely block area that had not been exposed to UV light. In contrast, in the SIMs fabricated with pps = 800 and 1000 μm, the pores were clearly formed (Fig. 3g,h). SIM with pps of 800 μm presents waffle-shaped patterns with a straight frame part and a square-shaped base part. In the SIM with pps = 1000 μm, the frame was blunt and the base was narrow and circular. The average thicknesses of the frame and base parts increase with decreasing pps (Fig. 3i), which indicates degree of exposure of the pre-gel solution to UV light, and therefore the degree of polymerization increases as pps decreases.

The frame, base, bottom, and pores were fabricated concurrently in the membrane after a single UV light illumination for 4 min by using a photomask with dual pattern sizes of 1 and 0.6 mm (Fig. 3j). At pps = 1000 μm, the frame and base parts formed between the patterns, and the pores were clearly formed. The thin bottom part is formed under 600 μm of pps. Each part is clearly distinguished by noting the inflection points of light intensity across the membrane (Fig. 3k). The black background shows through the pore, so the light intensity was lowest at the pore. The thinnest bottom part of the membrane was semi-transparent, and the highest light intensity was detected in the frame part.

### Thermal response of SIMs

**Figure 4:**
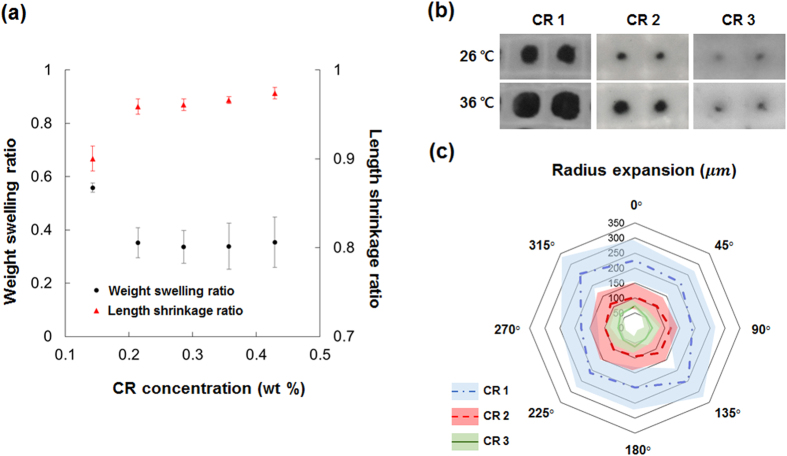
(**a**)Variations in the weight swelling ratio and length shrinkage ratio of the PNIPAAm membrane according to initial crosslinker concentration. **(b)** Thermal response of SIMs. Final equilibrium states of the three SIMs at initial 26 °C(upper) and 36 °C(lower). **(c)** Star chart of degrees of radius expansion (lines) and standard deviations (shaded) of pores in the three SIMs at intervals of 45°.

To examine the effect of crosslinking level on thermal response of the fabricated membranes, five different PNIPAAm membranes were fabricated with CR 1 to 3 pre-gel solutions (Table S1). The weight swelling ratio, which indicates the degree of water extrusion from the polymer, and the length shrinkage ratio, which represents the deformability of the polymer membrane, were also measured. The weight swelling ratio decreased, but the length shrinkage ratio of the corresponding hydrogels increased as the crosslinker concentration increased from 0.14 wt% to 0.43 wt% (Fig. 4a). This finding implies that the water-holding capacity and deformability decreased as crosslinker concentration increased. When the weight percentage of the crosslinker was > 0.2, the two ratios were insensitive to crosslinker concentration.

To analyze the thermal response of the SIMs fabricated with various crosslinking levels, SIMs fabricated with CR 1, 2, and 3 pre-gel solutions were heated at temperatures higher than the lower critical solution temperature (LCST, 32 °C). Heating of SIMs causes polymer shrinkage, which induces membrane deformation. During heating, the edge of the membrane was anchored to the glass substrate; therefore, the highly-crosslinked frames deformed slightly while maintaining the stability of the entire structure. However, moderately-crosslinked base parts deformed greatly and pore sizes varied^25^.

The initial (26 °C) and final (36 °C) equilibrium states differed among the three SIMs (Fig. 4b). At 26 °C, pores in the SIMs fabricated with CR 1, 2, and 3 solutions were of different sizes under the same fabrication conditions. The degrees of radius expansion also differed according to the crosslinking level; the pore radius of the SIM with CR 1 expanded to some extent, whereas that of CR 3 changed minimally. The degrees of radius expansion of the pores were quantitatively measured, and the results were depicted with an angle interval of 45° (Fig. 4c). The average radius expansions of SIMs fabricated with CR 1, 2, and 3 solutions were 251.6 ± 25.9 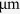, 106.4 ± 8.8 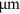, and 59.4 ± 7.1 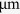, respectively. Thus, low crosslinking levels produce SIM with greatly-deformable pores.

### Transparency of SIMs

**Figure 5:**
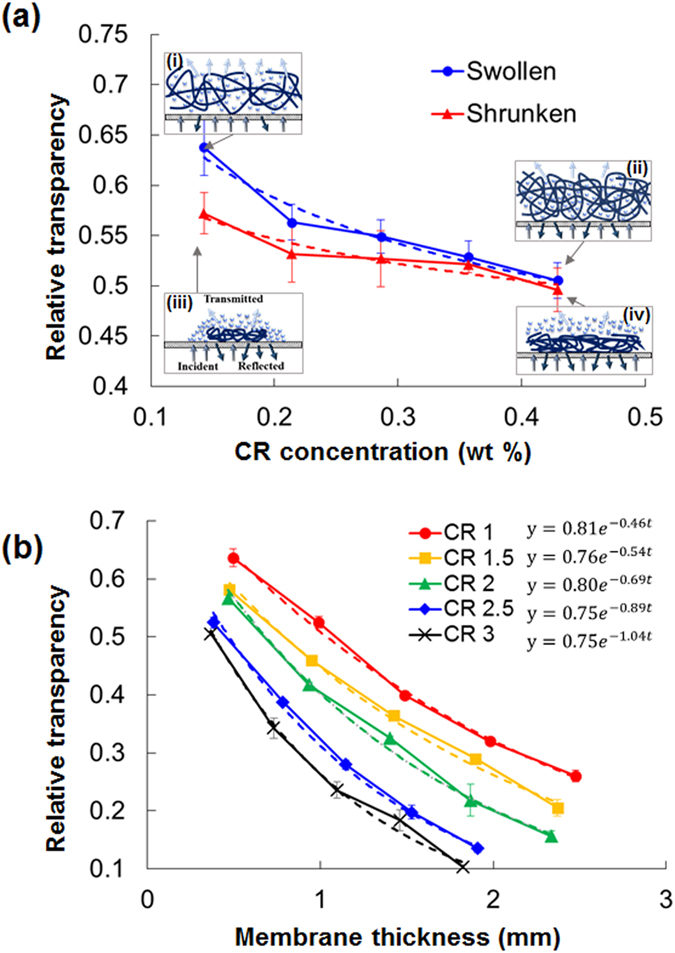
(**a**) Variation in the relative transparency of the PNIPAAm membranes at the swollen and shrunken states depending on the initial crosslinker concentration of the pre-gel solutions. (Inset) Schematic of light transmitted through the moderately and highly crosslinked polymer membranes at the swollen (26 °C) and shrunken (36 °C) states. **(b)** Variations in the relative transparency of the PNIPAAm membranes in the swollen state vs. membrane thickness.

As polymerization progresses, a certain amount of light passing through a membrane is lost by the polymer chains, and causes membrane to become opaque. The opacity of the fabricated PNIPAAm membranes is influenced by the crosslinking level in the membrane. The relative transparency of the PNIPAAm membranes was estimated based on transmitted light intensity by varying the initial crosslinker concentration of the pre-gel solutions. The relative transparency of the membrane decreased with increasing crosslinking level when the membrane was in the swollen state at 26 °C (Figs 5a and S3). The membrane fabricated with CR 1 was 1.26 times more transparent than the membrane with CR 3.

After heating at temperatures higher than LCST, the relative transparency of the shrunken membranes was less sensitive to changes in crosslinker concentration compared with that of the swollen membranes. The relative transparency of the shrunken membrane with CR 1 was 1.15 times higher than that of the membrane with CR 3. Given that PNIPAAm is a translucent material, some incident lights is reflected, absorbed, or scattered, whereas the rest are transmitted. In general, upon striking a material, light bounces back into all directions because of the irregularities and complexity of the polymer chains of the material. In the swollen state (temperature < LCST), the densities of the moderately and the highly crosslinked membranes were distinct; therefore the transmitted light intensity differed [insets (i, ii)]. As the degree of crosslinking in the polymer increases, the polymer chains become intricately entangled. Therefore, incident light is easily reflected or scattered. Meanwhile, when the membranes are shrunken (temperature > LCST), the polymer chains in the membrane densely collapse and extrude water molecules. Thus, the incident light cannot easily pass through the material, regardless of the level of crosslinking [insets (iii, iv)]. Overall, the difference in transparency of SIMs between the swollen and shrunken states decreases with increasing crosslinker concentration.

The transparency of the PNIPAAm membranes decreased exponentially as membrane thickness increased (Fig. 5b), but the exponential index of the decreasing trend line increased as the crosslinking level increased; this change indicates that a highly-crosslinked polymer membrane can block more light than a moderately-crosslinked one. The relative transparency varied within the range of 0.03–0.05/100 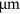 as the crosslinker concentration increases from 0.14 wt% to 0.43 wt%. Crosslinkers redistribute throughout the SIM by polymerization-induced diffusion. In addition, the frame, base, and bottom parts of the SIM contain different amounts of crosslinker. Therefore, the three parts transmit different amounts of light.

**Figure 6:**
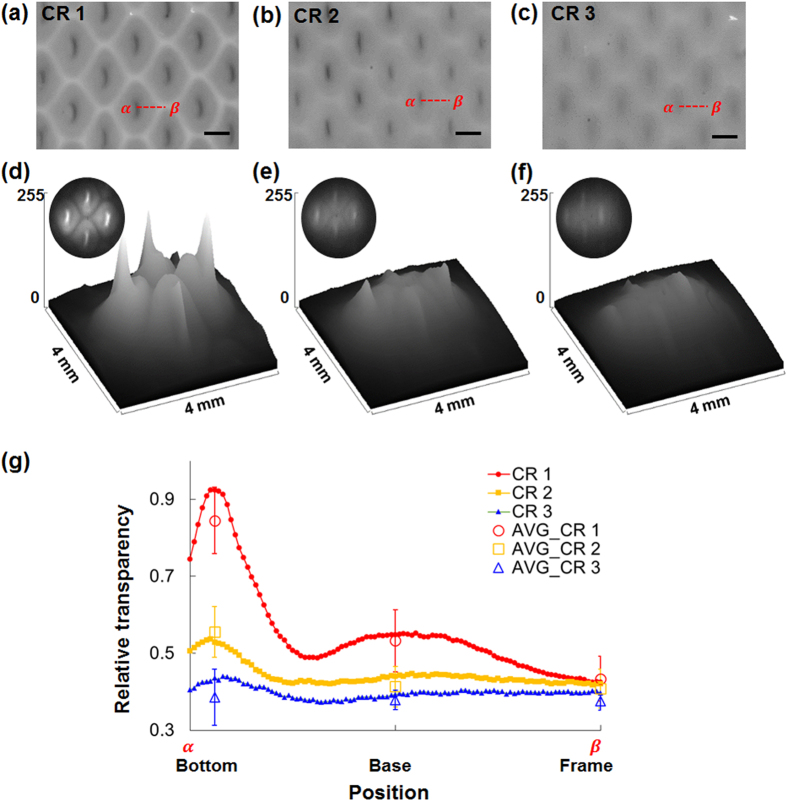
(**a–c**) Optical images of the membranes fabricated with CR 1–3 pre-gel solutions. **(d–f)** Surface plots of light intensity transmitted through the SIMs. **(g)** Variations in relative transparency of the SIMs across the dotted lines (α–β) in (**a–c**). Hollow points: average relative transparency at each part of the SIM. Scale bar: 1 mm.

A photomask with slit patterns of 175 μm width and 1 mm length was used to create a triple-parted membrane using CR 1, 2, and 3 pre-gel solutions. In optical images (Fig. 6a–c), the frame, base, and bottom parts are clearly distinguished when the CR 1 solution used, but not when CR 2 or CR 3 was used. The light intensity transmitted through the corresponding SIMs decreased as crosslinking increased (Fig. 6d–f). In the SIM fabricated with CR 1, the transmitted light intensity varied significantly among regions; the thick frame part, which is highly crosslinked, transmitted relatively little light, and the bottom part, which is moderately crosslinked transmitted the most light. In the SIM fabricated with CR 2, similarly, the transmitted light intensity also varied among regions. However, SIM fabricated with CR 3 transmitted little light intensity over the whole structure.

Relative transparency was measured along lines from the center of the aperture to its frame (Fig. 6g). The relative transparencies at the frame were all similarly low, regardless of crosslinker concentration. In all cases, the thinnest bottom part was most transparent. As the crosslinker concentration increased from CR 1 to CR 3, the fluctuations in the relative transparency decreased. The bottom of the SIM with CR 1 was about 1.95 times more transparent than the frame and 1.23 times more transparent than the base. In the SIM made using CR 2, bottom was 1.36 times more transparent than the frame and 1.01 times more transparent than the base; in the SIM made using CR 3, the bottom was 1.03 times more transparent than the frame and 1.01 times more transparent than the base.

### Feasible applications

**Figure 7:**
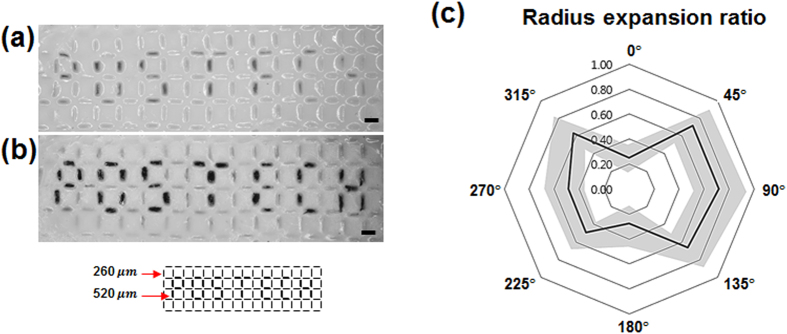
SIMs with inscribed letters were fabricated with CR 3 pre-gel solution by using the photomask shown in the inset. **(a)** Optical images of the fabricated SIM. The bottom part was fabricated under a pattern of 260 μm width, and the pores were formed under the pattern of 520 μm width. **(b)** Optical image of the membrane heated to temperatures higher than LCST; the pores opened to show letters “POSTECH” clearly. **(c)** Radius expansion ratios of vertical slit pores over angle intervals of 45°. Scale bar: 1 mm.

A triple-parted membrane with completely blocked pores or partially blocked pores can be fabricated by changing the photomask pattern (Fig. 3j). Changes in pre-gel solution composition, especially with varied crosslinker concentration, may also alter both the morphology of the SIM and the transparency of each part of the membrane. To demonstrate these effects, a polymer membrane with inscribed letters was fabricated (Fig. 7a,b) using CR 3 pre-gel solution through the photomask shown in the inset. The photomask consists of slits of 1 mm length and 260 μm width of background and seven segment letters with 520 μm width. The bottom part (Fig. 7a) was fabricated under the pattern of 260 μm width, and the pores were formed under the pattern of 520 μm width. When the membrane was heated at temperatures higher than the LCST, only the pores open and expand into an elliptical configuration, and the letters “POSTECH” appeared clearly (Fig. 7b). The membrane reversibly returns to the original swollen state after cooling at temperatures lower than the LCST. The radius expansion ratios *r*_α_ of vertical slit pores were assessed over angle intervals of 45° (Fig. 7c). The average radius expansion ratios of *r*_0°_(=0.25) and *r*_180°_(=0.27) are relatively lower than those of other directions whose average value is approximately 0.62.

## Conclusion

In this study, a novel SIM was fabricated using temperature-responsive PNIPAAm by effectively exploiting polymerization-induced diffusion on a macro-scale surface. Diffusion induced by chemical potential gradient allows the monomers and crosslinkers of the pre-gel solution to accumulate in the region illuminated by high-intensity UV light, thereby accelerating polymerization. Free radicals disperse radially to the unexposed area, and polymerization decelerates at the edges of the mask patterns. At this point, the diffused free radicals disperse toward the unexposed area initiate polymerization even under the patterns not directly reached by UV light; as a result, a third part of the membrane formed. Consequently, a three-part polymer membrane with size-controllable pores is fabricated with single UV light exposure. Various triple-parted membranes with completely-blocked or partially-blocked pores can be easily fabricated by changing the photomask design and composition of the pre-gel solution. Moreover, the morphological configuration, as well as the deformability and transparency of the SIM, can be easily manipulated.

Among the multi-sensitive responses of guard cells to various environmental stimuli (changes in temperature, pH, humidity, light intensity, etc), the fabricated SIMs mimic the thermal responsiveness of guard cells. Similar to guard cell movement in plant leaves, the fabricated SIMs can regulate the opening/closing of pores by swelling or shrinking thermally-responsive hydrogels. Moreover, similar to stomatal patchiness, which represents heterogeneous stomatal apertures on a leaf, the penetrated pores in the SIM open their aperture completely, whereas the incompletely-penetrated pores do not show any opening behavior. Thus, the SIM fabricated in this study is a smart membrane that can automatically sense and react to temperature changes by using thermal responsiveness. Therefore, the developed stimulus-responsive SIM is unquestionably novel and has strong potential for various engineering applications in the future.

## Methods

### Synthesis of SIM

Photo-crosslinkable poly(*N*-isopropylacrylamide) (PNIPAAm) was synthesized by free-radical polymerization to fabricate the SIM. A pre-gel solution was prepared by dissolving 100 mg of *N*-isopropylacrylamide monomer (NIPAAm, Sigma Aldrich) in 0.7 ml of deionized water. Then *N*,*N*′-methylenebisacrylamide (1, 1.5, 2, 2.5, or 3 mg MBAm; Sigma Aldrich) and 2-hydroxy-1-[4-(hydroxyethoxy)phenyl]-2-methyl-1-propanone (1 mg Irgacure 2959; Sigma Aldrich) were added to the monomer solution as a crosslinker and a photoinitiator, respectively (Fig. S1, Table S1). Various patterns were designed and printed on the transparent photomasks which is made of polyester. The pre-gel solution was poured into the space between the photomask and the dish. The solution was then irradiated for 4 min by UV light (VIRVER Lourmat-4.L, France; *λ* = 365 nm, 4 W) (Fig. 1a).

Free radicals were generated in the illuminated regions to initiate polymerization, and those produced in the irradiated region diffused toward the unexposed regions under the mask patterns. The crosslinkers, which were rapidly consumed in the exposed area during polymerization, diffused from the unexposed area toward the exposed area, depending on the chemical potential gradient in the polymerizing medium (Fig. 1b). During polymerization, the monomers and crosslinkers recrystallized into a network, and the cross-linked PNIPAAm covalently attached to the photomask substrate (Fig. 1c,d)^33^,^34^. The fabricated three-part (frame, base, bottom) SIM was carefully peeled from the photomask and stored in distilled water to fully hydrate the membrane and dissolve excess reactants (Fig. 1d).

### Morphological structure of SIM

The surface morphologies of the fabricated SIMs were observed using a stereoscope (Stemi 2000c; Zeiss, Germany) attached to a digital camera (NIKON D700). The gels were adsorbed by the cationic dye rhodamine 6G. The 3D morphological structures of the SIMs were observed using a Leica-SP5 confocal microscope attached with a 5× objective lens. The captured images were post-processed using the Leica software. The 3D configuration of each SIM was reconstructed using the Amira^®^ software (Visualization Science Group). The thicknesses of the frame, base, and bottom of the SIMs were measured from the captured cross-sectional images.

### Measurement of structural characteristics

PNIPAAm shrunk at temperatures higher than the LCST but swelled at temperatures lower than the LCST. The PNIPAAm membranes were fabricated with CR 1–3 pre-gel solutions to examine the effect of crosslinker concentration on the thermal response of PNIPAAm used in the present system. The temperature of the freely suspended PNIPAAm membrane was increased from 26 °C to 36 °C at a rate of 3 °C/min by using a Peltier chip to shrink the polymer network. The weight swelling ratio is defined as 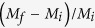, where 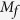 and 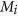 are the final and initial masses of the membrane, respectively. The length shrinkage ratio is defined as 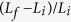, where 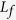 and 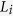 are the final and initial lengths of the membrane, respectively.

The edges of SIMs fabricated with CR 1, 2, and 3 were anchored to a glass substrate with adhesive glue and then heated. The images of the SIMs deformed by temperature increase were captured using a digital camera (NIKON D700) attached to a stereoscope. The radius expansion ratio 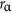 of the pores in each SIM is defined as 
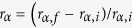
, where 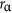 is the radius expansion ratio along the 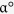 axis and 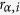 and 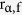 represent the initial and final radii along the 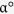 axis, respectively. The radius expansion ratios of the SIM pores were measured at an angle interval of 45°.

### Measurement of transparency

A 120 W metal halide lamp (X-Cite 120XL) was used to illuminate the test samples. Samples were placed on the inverted microscope (Zeiss Axiovert 200), and the light transmitted through them was captured by a Photron APX camera attached with 2.5×/10× objective lens (Zeiss Plan–Neofluar) (Fig. S2). In the measurement region of interest (ROI), the incident light was nearly homogeneous, and the scattering effect was negligible. The relationship between measured light intensity and material properties can be expressed by the Beer–Lambert law^38^:


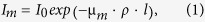


where 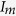 is the average light intensity of the images transmitted through the membrane, 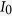 is the initial undisturbed intensity of the incident beam, 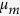 is the opacity of the test sample, and 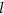 is the path length of the light propagating through the test sample. Using the definition of density 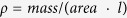, Equation (1) was rewritten as 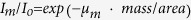. Given that the measured ROI is fixed, the value of (mass/area) can be considered as constant. The opacity 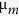 of each membrane can be evaluated vs. the opacity 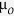, where the relative transparency is defined as follows:


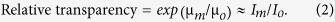


## Additional Information

**How to cite this article**: Kim, H. and Lee, S.-J. Fabrication of Triple-parted Stomata-inspired Membrane with Stimulus-responsive Functions. *Sci. Rep.* **6**, 21258; doi: 10.1038/srep21258 (2016).

## Supplementary Material

Supplementary Information
