# Supplementary material for: Fabrication of Triple-parted Stomata-inspired Membrane with Stimulus-responsive Functions
Source: Sci Rep. 2016 Feb 18;6:21258. doi: 10.1038/srep21258 (PMC4757937; doi:10.1038/srep21258)
Supplement: Supplementary Information [file srep21258-s1.pdf]

Scientific report

Supplementary information

Fabrication of Triple-parted Stomata-inspired Membrane with Stimuli-responsive Functions

Hyejeong Kim[a], and Sang-Joon Lee[a,*]

[a] Center for Biofluid and Biomimic Research, Department of Mechanical Engineering, Pohang University of Science and Technology (POSTECH), Pohang, 790-784, South Korea.

* Corresponding author.

Tel.: +82 54 279 2169; Fax: +82 54 279 3199.

E-mail address: sjlee@postech.ac.kr (S.J. Lee)

Supplementary

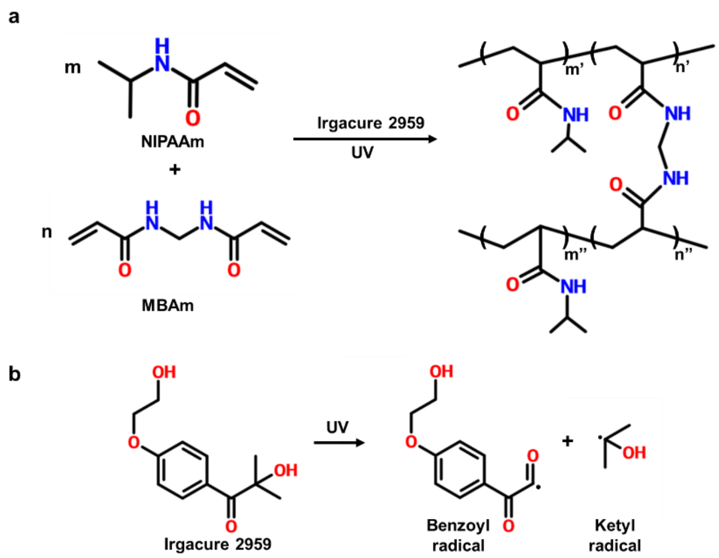

Figure S1. (a) Schematic of PNIPAAm hydrogel synthesized through free radical copolymerization with MBAm as a crosslinking agent. (b) Schematic of free radical formation with photoinitiator illumination.

#	NIPAAm	Irgacure 2959	MBAm	CR (wt %)
CR 1			1 mg/ 100 μl	0.143
CR 1.5			1.5 mg/ 100 μl	0.214
CR 2	100 mg/ 500 μl	1 mg/ 100 μl	2 mg/ 100 μl	0.286
CR 2.5			2.5 mg/ 100 μl	0.357
CR 3			3 mg/ 100 μl	0.429

Table S1. Chemical composition of five different pre-gel solutions. The pre-gel solutions contain the same amount of monomers and photoinitiator but different crosslinker (MPAm) contents.

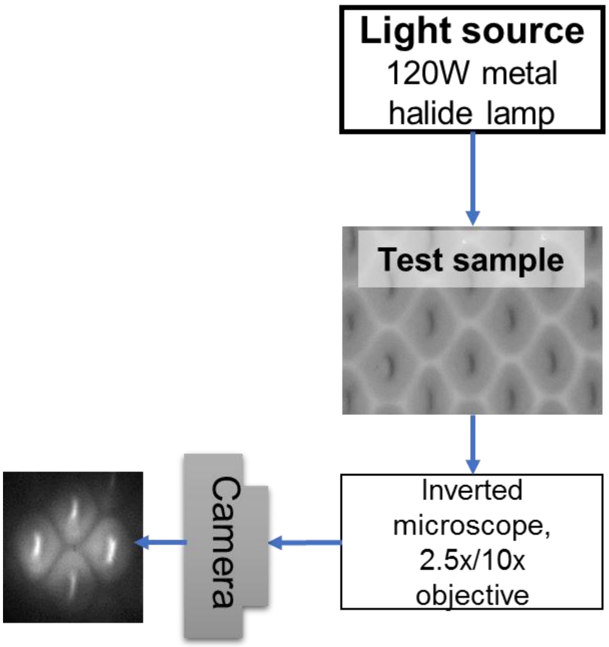

Figure S2. Schematic of the experimental setup for light intensity measurement. Light from a 120 W metal halide lamp is illuminated to the test samples. The light transmitted through the samples is detected by an inverted microscope equipped with a Photron APX camera.

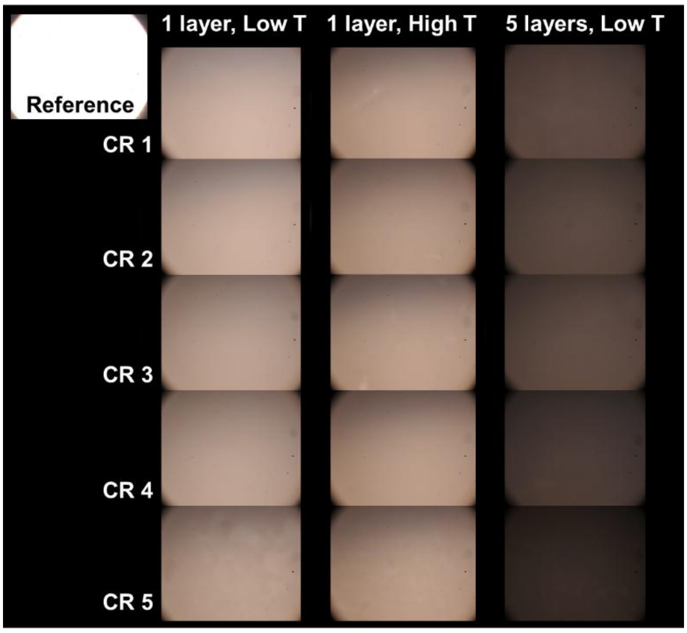

Figure S3. Optical images of SIM fabricated with five different pre-gel solutions (CR 1–CR 3). The relative opacity of the membrane is evaluated from the light intensity transmitted through the membranes. The transmitted light intensity decreases with increasing crosslinking level and membrane thickness.
